# A Compartmental Comparison of Major Lipid Species in a Coral-*Symbiodinium* Endosymbiosis: Evidence that the Coral Host Regulates Lipogenesis of Its Cytosolic Lipid Bodies

**DOI:** 10.1371/journal.pone.0132519

**Published:** 2015-07-28

**Authors:** Hung-Kai Chen, Shin-Ni Song, Li-Hsueh Wang, Anderson B. Mayfield, Yi-Jyun Chen, Wan-Nan U. Chen, Chii-Shiarng Chen

**Affiliations:** 1 Department of Marine Biotechnology and Resources, National Sun Yat-Sen University, Kaohsiung, 804, Taiwan; 2 Graduate Institute of Marine Biology, National Dong-Hwa University, Pingtung, 944, Taiwan; 3 Taiwan Coral Research Center, National Museum of Marine Biology and Aquarium, Pingtung, 944, Taiwan; 4 Living Oceans Foundation, Landover, MD, 20785, United States of America; 5 Department of Biological Science and Technology, I-Shou University, Kaohsiung, 805, Taiwan; Scottish Association for Marine Science, UNITED KINGDOM

## Abstract

The lipid body (LB) formation in the host coral gastrodermal cell cytoplasm is a hallmark of the coral-*Symbiodinium* endosymbiosis, and such lipid-based entities are not found in endosymbiont-free cnidarian cells. Therefore, the elucidation of lipogenesis regulation in LBs and how it is related to the lipid metabolism of the host and endosymbiont could provide direct insight to understand the symbiosis mechanism. Herein, the lipid composition of host cells of the stony coral *Euphyllia glabrescens*, as well as that of their cytoplasmic LBs and *in hospite Symbiodinium* populations, was examined by high performance liquid chromatography (HPLC) and gas chromatography/mass spectrometry (GC/MS), and six major lipid species were identified: wax esters, sterol esters, triacylglycerols, cholesterols, free fatty acids, and phospholipids. Their concentrations differed significantly between host coral cells, LBs, and *Symbiodinium*, suggesting compartmental regulation. WE were only present in the host coral and were particularly highly concentrated in LBs. Amongst the four species of WE, the monoene R = C18:1/R = C16 was found to be LB-specific and was not present in the host gastrodermal cell cytoplasm. Furthermore, the acyl pool profiles of the individual LB lipid species were more similar, but not equal to, those of the host gastrodermal cells in which they were located, indicating partially autonomous lipid metabolism in these LBs. Nevertheless, given the overall similarity in the host gastrodermal cell and LB lipid profiles, these data suggest that a significant portion of the LB lipids may be of host coral origin. Finally, lipid profiles of the *in hospite Symbiodinium* populations were significantly distinct from those of the cultured *Symbiodinium*, potentially suggesting a host regulation effect that may be fundamental to lipid metabolism in endosymbiotic associations involving clade C *Symbiodinium*.

## Introduction

Hermatypic corals have long been shown to perform endosymbiotic and mutually-benefit association with the dinoflagellate *Symbiodinium* (i.e. the endosymbiont) in the host gastrodermal cells [1, 2]. Nevertheless, the dual-compartmental metabolism between the host gastrodermal cells and the dinoflagellate populations residing within them has been greatly understudied. Although it is evident that the coral hosts acquire some metabolic capabilities via this symbiosis, such as photosynthesis and essential amino acid synthesis [3], the molecular regulation of these acquisitions remains undescribed.

Patton *et al*. provided the first evidence to show that *Symbiodinium* primarily performed the role of lipid synthesis in corals via the metabolism of host-derived acetate [4]. It was also postulated that *Symbiodinium* are likely to synthesize fatty acids (FA) from photosynthate and return these lipids to their hosts in the form of wax esters (WE) and triacylglycerides (TAG). This was confirmed later on that lipids in the host gastrodermal cells are mainly WE (22–49%) and triglycerides (18–37%) [5–6]. How the lipids actually flow between compartments is currently unclear, though an assessment of unique features of endosymbiotic coral gastrodermal cells known as lipid bodies (LB) may help to elucidate this matter.

Since the biogenesis of host coral intracellular LBs is dependent upon the presence of *Symbiodinium*, these oil droplet-like entities have led to several studies aiming to understand the endosymbiotic mechanism in corals [7–9]. Muscatine *et al*. [10] were the first to note that *Symbiodinium* secrete “lipid droplets” (from henceforth referred to as LBs) *in hospite*. In other organisms, LBs are formed from neutral lipids that are synthesized between the leaflets of the membranes of the endoplasmic reticulum (ER), and mature LBs later bud from the ER to form independent organelles containing a monolayer of phospholipids with a unique FA composition [11]. In the hermatypic coral *Euphyllia glabrescens*, proteomic and morphological examinations [8] indicated that, in contrast to Muscatine *et al*. [10], both *Symbiodinium and* ER in coral gastrodermal cells, might involve in LB biogenesis. Moreover, coral LB density, growth, composition, and ultrastructure all demonstrate diel rhythmicity [9]. As a consequence, the LB formation in coral host seems to be not only related to lipid flow in the host-endosymbiont compartments, but also reflect the endosymbiotic status.

Animals lack the enzymes required to introduce double bonds into fatty acids beyond the Δ9 position, which is necessary for the n-3 and n-6 pathways [12]. Papina *et al*. [13] found FA specific to *Symbiodinium* (*e*.*g*., 18:4n-3, 22:5n-3, and 22:6n-3) in the host gastrodermal cells, suggesting that *Symbiodinium* not only provide their coral hosts with saturated FA, but also with a diverse array of polyunsaturated FA (PUFA). The translocation of FA from the *Symbiodinium* populations to their coral hosts dramatically influences the hosts’ FA pools [14], and the composition of these pools could be a diagnostic indicator of coral health. This hypothesis was confirmed by verifying that FA composition changes during bleaching, in which *Symbiodinium* are lost and harmful bacterial densities oftentimes increase [15].

In contrast, less is known about how the host coral modifies the lipid composition and metabolism of its *in hospite Symbiodinium*. In the present study using HPLC and GC/MS, we purified and identified FA pools from three different cellular compartments of the coral *E*. *glabrescens*: the host coral gastrodermal cell cytoplasm, the host coral LBs, and the *in hospite Symbiodinium* populations. It was hypothesized that the host coral, rather than the endosymbionts, actually contributes the majority of the lipids to their gastrodermal LBs. By looking at lipid species distribution and concentration in individual fractions of the coral holobiont, the ultimate compartment of origin of the lipid constituents of the host cell gastrodermal LBs could be verified.

## Materials and Methods

### Coral collection/maintenance and *Symbiodinium* culture

Colonies of *E*. *glabrescens* were collected by SCUBA divers from the inlet of Taiwan’s third nuclear power plant (21°57.376'N, 120°45.291'E) at depths of 3–8 m in Nanwan Bay, the southernmost embayment in the mainland region of the country. The coral collection was approved by the Kenting National Park Management Office in Taiwan. Colonies were placed in an upright position in a 4 kl outdoor tank with flow-through seawater (exchange rate = 80 L/h) and maintained under a natural photoperiod (12L:12D) with additional air circulation in the husbandry center of the National Museum of Marine Biology and Aquarium (NMMBA). A microprocessor-controlled cooler (First FC-45, Aquatech, Kaohsiung, Taiwan) was connected to the tank, and the temperature was maintained at 26.5±1°C (standard deviation). Colonies were exposed to natural, though partially shaded, light. Ten tentacles (stretched length of ~3 cm) were amputated from polyps of eight *E*. *glabrescens* colonies using curved surgical scissors, randomly mixed in a seawater table, sorted into four groups of 20 tentacles to serve as the four biological replicates for downstream analyses, and then transferred to the laboratory and washed with filtered seawater (FSW) for further use.

The cultured *Symbiodinium* (clade C) used in this study was purchased from the Center for Culture of Marine Phytoplankton (strain CCMP 2466; West Boothbay Harbor, ME, USA). The cells were cultivated in 2% Guillard’s f/2 medium [16] in artificial seawater (ASW) containing 100 U mL^−1^ penicillin and 100 μg mL^−1^ streptomycin at RT under a photosynthetically active radiation (PAR) level of 40 μmol m^−2^ s^−1^ across a 12L:12D cycle in a Model LTI-613 growth chamber (Taiwan Double Eagle, Co., Ltd., Taipei, Taiwan).

### Tissue fractionation to separate three cellular compartments (host gastrodermal cell lysates, host LBs, and *in hospite Symbiodinium*)

#### The gastrodermal separation and homogenization

Eighty tentacles of *E*. *glabrescens* were amputated and collected. Tentacle tips were removed using microscissors (Spring Type, AESCULAP, Center Valley, PA, USA) to prevent interference from the nematocytes during the experimental process. The gastroderms were then separated from the epiderms by 3% *N*-acetylcysteine (pH 8.2) treatment as previously described [16]. After subsequent incubation in 2 mL of ASW containing 1X complete protease inhibitor cocktail (Roche, Madison, WI, USA), the gastroderms were homogenized on ice with ten passes of a 7-mL glass tissue grinder (Kimble/Kontes, Vineland, NJ, USA). The crude homogenate was then passed through a syringe (23G×1 (1/4)", Top Surgical, Taiwan) 15 times, to lyse gastrodermal cells and free intact host LBs and *Symbiodinium* into the solution. The homogenates were then centrifuged (500×g at 4°C for 5 min) to separate the LB- and host cell lysate-containing supernatant from the pelleted *Symbiodinium* with host cell debris.

#### Purification of *in hospite Symbiodinium* and host LB

The pellets containing *Symbiodinium* and decries of gastrodermal cells were separated by discontinuous sucrose gradient centrifugation (0%-25%-40%-50% sucrose, 10,000×g at 4°C for 5 min). *Symbiodinium* were collected from the interfaces of 40%-50% and 25%-40%. The presence of *in hospite Symbiodinium* was further confirmed via light microscopy. The cells were then washed twice with 0.5 mL ASW, centrifuged at 500×g (4°C for 5 min), and re-suspended in ASW at an approximate concentration of 6×10^5^ cells∙mL^-1^. As an experimental control, cultured *Symbiodinium* maintained at the exponential growth stage (6×10^5^ cells∙mL^-1^) were also collected from each of four 160-mL cultivation bottles.

LBs were purified by two stepwise sucrose gradient ultracentrifugations (0.4–0.66 M and 0–0.36 M). First, the LB-containing supernatants (~1.8 mL) from the homogenization step were mixed with ice-cold 1.2 M sucrose (2.2 mL in PBS containing 1X protease inhibitor cocktail, pH 7.5) in a 12-mL ultracentrifugation tube (UltraClean tube, Beckman Coulter, Brea, CA, USA). The top of the solution was then overlaid with cold 0.4 M sucrose (6 mL in PBS with 1X protease inhibitor cocktail, pH 7.5). After centrifugation at 150,000×g at 4°C for 60 min using a swing rotor SW-41, Optima L-100 XP Ultracentrifuge (Beckman Coulter), the top layer containing LBs was collected and deemed the ‘‘buoyant crude LBs” fraction (1.6 mL). The buoyant crude LBs were then incubated with cold 0.33 M sucrose (2.4 mL in PBS plus 1X protease inhibitor cocktail) containing Tween-20 (final concentration = 0.04%; Sigma-Aldrich) at 4°C for 10 min to remove cellular materials attached to the outside of the LBs. Afterwards, they were transferred to a new ultracentrifugation tube and overlaid with 6 mL of cold PBS (plus 1X protease inhibitor cocktail) for the second stepwise sucrose gradient ultracentrifugation (0–0.36 M top-bottom; 150,000×g at 4°C for 30 min). The top layer containing LBs was collected and deemed the ‘‘buoyant, detergent-washed LBs.”

#### Purification of host gastrodermal cell lysates

The gastrodermal cell lysates were collected from two fractions. One fraction was collecgted from the 0%-25% interface of the discontinuous sucrose gradient centrifugation step (i.e. 0%-25%-40%-50% sucrose, 10,000×g at 4°C for 5 min). The second fraction was from the 8.4 mL of solution that remained after removing the buoyant crude LBs fraction (1.6 mL) from the first sucrose gradient ultracentrifugation (0.4–0.66 M, 150,000×g at 4°C for 60 min).

### The purity assessment for gastrodermal cell lysates, host LBs, and *in hospite Symbiodinium*

To evaluate the purity of the gastrodermal cell lysates, LBs, and *in hospite Symbiodinium* fractions, analyses of western blotting were conducted to demonstrate an absence of contamination of host and *Symbiodinium* proteins from the non-targeted fractions (verified by Western blotting). Purity was verified as described previously [8], but with several modifications. First, the lysed host gastrodermal cells, LBs, and *Symbiodinium* (both *in hospite* and cultured) were delipidated according to the procedure described by Mastro and Hall [17]. Briefly, a ‘‘delipidation solution” (tributyl phosphate:acetone:methanol, 1:12:1, v/v/v) was added to the collected fractions at a 14:1 volume ratio on ice, followed by incubation at -20°C overnight. Precipitated proteins were then collected (3,202×g for 15 min at 4°C), washed sequentially with ice-cold methanol, tributyl phosphate, and acetone (30 min at 4°C for each wash), and vacuum-dried (5,000×g) at RT for 10–20 min. The precipitated proteins were then re-suspended in 1X SDS-PAGE sample buffer (62.5 mM Tris-HCl [pH 6.8], 2% SDS, 10% glycerol, and 10 mM DTT) and quantified with the 2-D Quant Kit (GE Healthcare, Piscataway, NJ, USA) according to the manufacturer’s recommendations.

Ten micrograms (10 μg) of each protein sample (n = 3 for each of the four fractions [host gastrodermal cell lysate, LBs, *in hospite*, and cultured *Symbiodinium*]) were subjected to 12% SDS-PAGE using a Bio-Rad (Hercules, CA, USA) electrophoresis unit (Mini PROTEAN 3 cell) [18]. Afterwards, the SDS-PAGE gel was equilibrated in Towbin buffer (25 mM Tris, 192 mM glycine, 20% MeOH, and 0.1% SDS, pH 8.0 [19]) and then blotted onto PVDF membranes (immobilon-PSQ 0.45 mm; Millipore, Germany) using the Bio-Rad Transblot apparatus (100 V for 2 h at 4°C). The membranes were incubated in blocking buffer (5% skim milk, 0.1% Tween-20, 100 mM Tris [pH 7.6], 150 mM NaCl) at RT for 1 h, followed by incubation with an antibody cocktail of rabbit anti-ribulose-1, 5-bisphosphate carboxylase/oxygenase (rubisco) large subunit (1:2,000 dilution; Cat. AS0037, Agrisera, Vannas, Sweden; the marker for presence of *Symbiodinium* proteins), mouse anti-actin (1:10,000 dilution; Cat. MAB1501, Millipore; the marker for presence of host coral and LB proteins, *sensu* [8]), and mouse anti-ADP-ribosylation factor (ARF) (1:500 dilution; Cat. Ab2806, Abcam, Cambridge, MA, USA; marker for host gastrodermal cell proteins only) in TBST buffer (0.1% Tween-20, 100 mM Tris [pH 7.6] and 150 mM NaCl) at 4°C overnight. The membranes were then washed five times with TBST buffer for 10 min each and incubated with HRP-conjugated goat anti-rabbit and anti-mouse IgGs (Millipore) in TBST buffer (1:5,000 dilution for each secondary antibody). The membranes were subsequently washed with TBST buffer, and the resulting proteins were visualized using the SuperSignal West Pico Chemiluminescent substrate kit (Cat. 34080, Thermo-Fisher Scientific, Waltham, MA, USA) according to the manufacturer’s recommendations.

### Lipid analyses

Total lipids from the three collected fractions and cultured *Symbiodinium* (n = 4 for each of the four treatments/compartments) were first extracted by the Bligh and Dyer procedure [20]. The extracted lipids were then analyzed by an HPLC instrument equipped with an evaporative light scattering detector (HPLC-ELSD) [21]. A Hitachi Model L7100 HPLC pump equipped with an auto-sampler (L7200, Hitachi, Japan) was used with a Sedex 80 ELSD (Sedere, France). The drift-tube temperature was maintained at 60°C, and the flow-rate of the nebulizer gas (nitrogen) was 2.5 kg/cm^2^. The detector response was quantified by electronic integration. Solvents were de-aerated with nitrogen gas. A column of YMC-PVA-SIL (10063 mm i.d.; 5 mm particles) was obtained from Hichrom Ltd. (Reading, UK).

The first solvent system individually fractionated seven class of lipids (a mixture of SE/WE, TAG, Col, FFA, phosphatidylethanolamine [PE], phosphatidylcholine [PC], and lyso-phosphatidylcholine [lyso-PC]) by retention time (ReT). This protocol required a quaternary gradient elution scheme consisting of n-hexane (Merck, Germany): petroleum ether (Merck) (2:8 v/v; solvent A), n-hexane (Merck): petroleum ether (Merck) (8:2 v/v; solvent B), propan-2-ol (Merck): acetonitrile (Merck): butan-2-one (Merck) (7:2:1 v/v/v; solvent C), and propan-2-ol:acetonitrile: butan-2-one: methanol (Merck): water: N-ethylmorpholine (Sigma-Aldrich, MO, USA): acetic acid (Merck) (56:14:7.2:14:8.4:0.42:0.15 v/v; solvent D) with the gradient elution program described in S1 Table.

Eight major lipid standards could be clearly separated and analyzed by this HPLC analysis, including WE (arachidyl dodecanoate, Sigma-Aldrich), SE (cholosteryl oleate, Sigma-Aldrich), an SE/WE mixture (ReT = 1.8 min), TAG (tricaprin; ReT = 5.2 min, Sigma-Aldrich), Col (“plant-derived;” ReT = 10.9 min, Avanti Polar Lipids, Inc., AL, USA), FFA (linoleic acid; ReT = 16.5 min, Sigma-Aldrich), PE (ReT = 31.9 min, Avanti Polar Lipids, Inc.), and PC (ReT = 35.3 min, Avanti Polar Lipids, Inc.), and Lyso-PC (18:1 Lyso-PC [1-oleoyl-2-hydroxy-sn-glycero-3-phosphocholine], ReT = 40.9 min, Avanti Polar Lipids, Inc.).

An SE/WE mixture was collected from first solvent system, and the second solvent system separated these two species by ReT. This step included a binary gradient elution scheme consisting of n-hexane (Merck): petroleum ether (Merck) (2:8 v/v; solvent A) and n-hexane (Merck): petroleum ether (Merck) (8:2 v/v; solvent B) with the gradient elution program described in S2 Table. The WE (ReT = 3.2 min) and SE (ReT = 4.3 min) lipid standards described above could be clearly separated and analyzed by this system.

Consequently, the integrated areas of the HPLC profiles were plotted as a function of lipid concentration, and 4–5 concentrations of each standard were used for each of the nine lipid species targeted herein: WE (1.95, 3.91, 7.81, 15.63, and 31.25 ng/μl), SE (1.95, 3.91, 7.81, 15.63, and 31.25 ng/μl), TAG (7.81, 15.63, 31.25, 62.5, and 125 ng/μl), Col (31.25, 62.5, 125, 250, and 500 ng/μl), FFA (31.25, 62.5, 125, 250, and 500 ng/μl), PE (62.5, 125, 250, and 500 ng/μl), PC (62.5, 125, 250, and 500 ng/μl), and Lyso-PC (62.5, 125, 250, and 500 ng/μl). Lipid species concentrations in the analyzed coral samples were then inferred from their integrated areas by solving for the best-fit linear regression equation: γ = aX+b, where γ = lipid concentration and X = integrated area.

### Gas chromatography/mass spectrometry (GC/MS)

In order to examine compositional differences in individual lipid species, which could provide direct identification of the acyl pools, FA moieties of specific lipid species were determined by lipid derivatization followed by GC/MS. The same lipid samples used above for HPLC analyses were converted to their constituent fatty acid methyl esters (FAME) by refluxing in 5 mL of a reagent consisting of concentrated sulphuric acid-toluene-methanol (1:10:20 v/v/v) and an internal standard (nervonic acid, Sigma-Aldrich) for 2 h at 90°C according to a published method [22]. After cooling, 5 mL of 5% sodium chloride and 5 mL of hexane were added. The hexane layer was recovered and added to 4 mL of 2% potassium bicarbonate. Finally, the samples were dried over anhydrous sodium sulfate, and the FAME were ready for injection.

FAME were analyzed on a gas chromatograph (GC, Varian CP-3800) and a mass spectrometer (Varian 320 MS) operated in full scan mode (scan range from 100 to 450 m/z). FA peaks were quantitatively and qualitatively analyzed using a 37-component FAME standard (Supelco, Sigma-Aldrich), stearidonic acid methyl ester (18:4n-3, Sigma-Aldrich), cis-7,10,13,16-docosatetraenoic acid methyl ester (22:4n-6, Sigma-Aldrich) and all-cis-7,10,13,16,19-docosapentaenoic acid methyl ester (22:5n-3, Sigma-Aldrich). WE peaks were identified using palmityl myristate (14:0/16:0), palmitate (16:0/16:0), oleate (18:1/16:0), and stearate (18:0/16:0) standards (LGC Standards, UK). The column for FAME was a CP-Sil88 capillary column of 20-m length and 0.25 mm i.d., and the stationary phase had a film thickness of 0.2 μm (Agilent Technologies, Inc., Santa Clara, USA). The column for WE was a VF-5ms capillary column (30 m length × 0.25 mm i.d., Agilent Technologies, Inc., Santa Clara, USA), and the stationary phase had a film thickness of 0.25 μm. Carbon dioxide was used as the carrier gas at a flow rate of 0.8 mL∙min^-1^. The temperature program was as follows: a CP-Sil88 column (for FMAE derivatives) was used at 50°C for 1 min, 50–200°C at 8°C min^-1^ for 5 min, 200–230°C at 20°C min^-1^, and a VF-5ms column (for WE) was used at 40°C for 1 min, 40–300°C at 25°C min^-1^ for 15 min, and 300–320°C at 2°C min^-1^ for 5 min. ReT data and mass spectra were compared against the NIST02 library (National Institute of Standards and Technology, Gaithersburg, MD, USA) to identify FA. Saturn GC/MS Workstation (ver. 6.9.3) software (Varian) was used to visualize spectra, integrate areas under peaks, and search the library. Peaks of the FA were identified, and the qantity of the individual FA species were calculated from a standard curve of five dilutions of the standards (FAME-37: 50, 100, 200, 400, and 800 ng/μl, WE standards: 3.13, 6.25, 12.5, 25, and 50 ng/μl) in an analogous matter as for the HPLC-based analyses. Concentrations of individual FA species were normalized with extracted sample proteins (μg). The relative protein ratio for a host gastrodermal cell/*Symbiodinium* cell/LB is ~5:5:1.

### Statistical analysis

All statistical analyses were performed using the Statistical Package for the Social Sciences (SPSS ver. 17.0, IBM, Armonk, NY, USA). The results were expressed as mean±SD (standard deviation), and tested for normality with Shapiro–Wilk’s test. In order to determine the effect of compartment (host gastrodermal cells, LBs, *in hospite Symbiodinium*, and cultured *Symbiodinium*), data were analyzed using Kruskal-Wallis tests given their tendency to lack normality, and Mann-Whitney U tests were used to determined individual mean differences; *p*<0.05 were considered to represent significant in each case. To portray correlations between FA moieties (ng/μg protein) in individual lipid species among host gastrodermal cells, LBs, and *Symbiodinium* and to evaluate the distribution of FAs in the samples, principal components analysis (PCA) was used.

## Results

### Methodological quality control and inter-compartmental lipid differences

**Fig 1:**
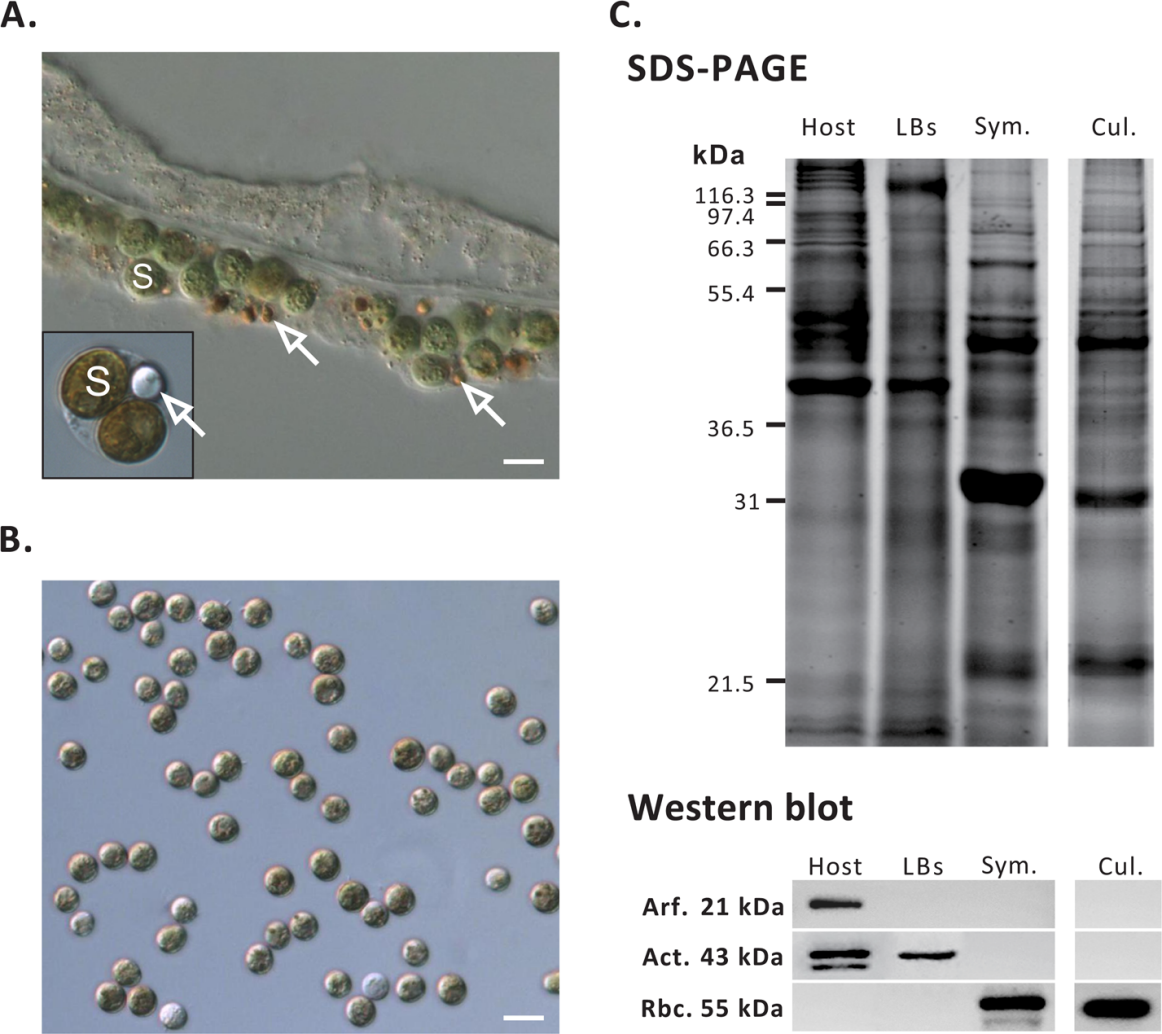
(A) Microscopic images of tentacle paraffin sections. Both *in hospite Symbiodinium* (S) and LBs (indicated by blank arrows) are visible in the gastroderm and an isolated symbiotic gastroderm cell (see the inset). (B) Cultured *Symbiodinium*. Scale bar, 10 μm. (C) The protein profiles and purity of LBs, host gastrodermal cell lysates (“host”), *in hospite Symbiodinium* (“Sym.”), and cultured *Symbiodinium* (“Cul.”) by SDS-PAGE and western blot examination. Arf, ADP-ribosylation factor; Act, actin; Rbc, rubisco.

Under DIC microscopy, LBs were shown to be opaque globules with an average diameter of ~3.5 μm while still within the gastroderm (Fig 1A). The exact location of LBs inside the symbiotic gastrodermal cell (SGC) is shown in the corresponding inset of Fig 1A, which represents a typical SGC. After gastrodermal separation, LBs were purified and separated from host gastrodermal cells and *Symbiodinium* (*sensu* Fig 1 and [8]). The purities of the separated fractions were assessed using antibodies specific for marker proteins for each compartment: rubisco for *in hospite* and cultured *Symbiodinium* (Fig 1B), animal actin for LBs and host cells, and ARF for the host cells only (Fig 1C). The detergent-washed LBs represent the pure LBs, as they expressed only an actin with a MW of 43 kDa (Fig 1C). In contrast, the host gastrodermal cell fraction contained a characteristic actin doublet and the ARF protein. *Symbiodinium* fractions were not associated with host or LB marker proteins and only expressed *Symbiodinium* rubisco (Fig 1C).

**Fig 2:**
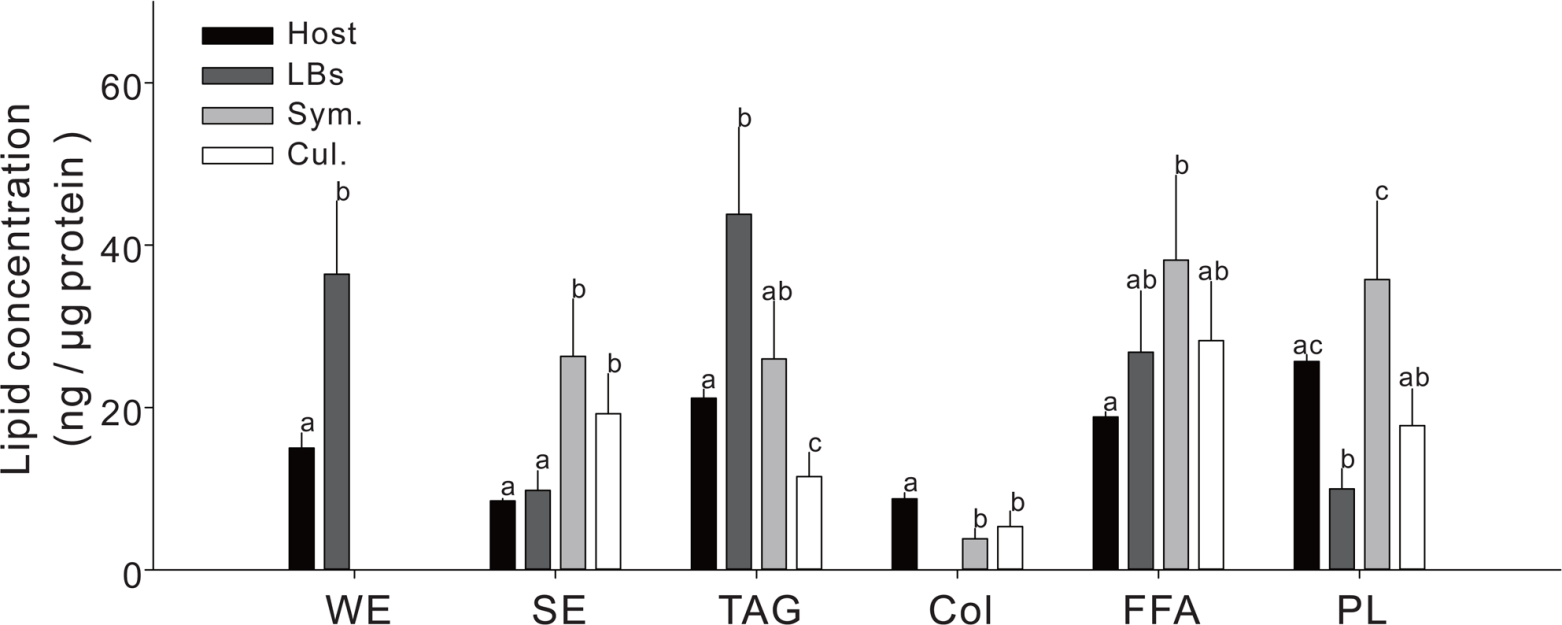
Concentrations of lipids in coral host gastrodermal cells (“host,” black columns), lipid bodies (“LBs”, dark gray columns), *in hospite Symbiodinium* (“Sym.”, light gray columns), and cultured *Symbiodinium* (“Cul.”, white columns). The full names of the individual lipid species can be found in the text. Values (mean±SD, n = 4) with different superscripts within a lipid species signify significant differences in concentrations between the four fractions (Mann Whitney post-hoc U tests, *p*<0.05). When a column is not depicted, the respective lipid was not detected.

Lipid species of purified LBs, host gastrodermal cells, and *Symbiodinium* (both cultured and *in hospite*) were quantified by HPLC (Fig 2). There were six major lipid species in the host cells, including WE, SE, Col, TAG, FFA, and PL. WE were only present in the host fractions (*i*.*e*., the host gastrodermal cells and host-derived LBs) and were highly concentrated in the LBs (36.4±9.0 ng/μg protein versus 15.0±1.8 ng/μg protein in host gastrodermal cells). WE were not detected in *in hospite* or cultured *Symbiodinium*. On the other hand, higher concentrations of SE were found in *in hospite* (26.3±7.0 ng/μg protein) and cultured *Symbiodinium* (19.2±4.9 ng/μg protein) relative to the host-derived fractions. Although Col concentrations were higher in host gastrodermal cells (8.8±0.7 ng/μg protein) than in *in hospite* and cultured *Symbiodinium* (3.8±1.3 and 5.3±1.9 ng/μg protein, respectively), no Col were measured in LBs. TAG, FFA, and PL were widely distributed in all fractions examined and were significantly concentrated in LBs (TAG: 43.8±10.7 ng/μg protein) and *in hospite Symbiodinium* cells (FFA: 38.1±10.4 ng/μg protein; PL: 35.8±9.6 ng/μg protein). Finally, the concentrations of all lipid species were higher *in hospite* versus cultured *Symbiodinium* (Fig 2) when normalized to total protein.

### Comparison of acyl chain concentrations in total lipid pools

The differences in the concentrations of lipid acyl chains between host gastrodermal cells, LBs, *in hospite Symbiodinium*, and cultured *Symbiodinium* are shown in Table 1. LBs possessed higher concentrations of 16:0 and 18:0 relative to host gastrodermal cells, *in hospite Symbiodinium*, and cultured *Symbiodinium*. Furthermore, the FA 20:0, 20:3n-6, 22:4n-6, and 20:4n-6 were only detected in LBs and coral host gastrodermal cells. LBs and *in hospite Symbiodinium* also contained 16:1n-7, which was undetectable in the host gastrodermal cells. The *in hospite* and cultured *Symbiodinium* were characterized by higher concentrations of 20:5n-3 than LBs and host gastrodermal cells. Finally the acyl chain 18:3n-6 was the most abundant lipid species in *in hospite Symbiodinium*.

**Table 1:** Concentrations of fatty acid acyl chains from host coral gastrodermal cells, LBs, *in hospite Symbiodinium*, and cultured *Symbiodinium*.

Acyl chain	concentration (ng/μg protein)				χ^*2*^ value	*p* value
	Host gastrodermal cells	LBs	*in hospite Symbiodinium*	Cultured *Symbiodinium*		
14:0	3.1 ± 1.9^**b**^	3.9 ± 1.2^**b**^	10.9 ± 1.2^**a**^	4.5 ± 1.2^**b**^	9.02	*
16:0	19.7 ± 1.2^**c**^	54.9 ± 4.3^**a**^	46.7 ± 1.2^**b**^	16.0 ± 1.4^**c**^	14.12	***
18:0	18.9 ± 0.5^**b**^	33.5 ± 2.9^**a**^	34.4 ± 2.9^**a**^	6.1 ± 1.3^**c**^	12.90	***
20:0	1.2 ± 0.6^**a**^	0.9 ± 0.3^**a**^	–	–	13.11	***
22:0	2.1 ± 0.7^**a**^	–	4.1 ± 1.0^**a**^	–	14.12	***
16:1 n-7	–	2.4 ± 0.5^**a**^	2.1 ± 0.8^**a**^	2.5 ± 0.4^**a**^	8.80	*
20:2 n-9	0.6 ± 0.3^**a**^	–	1.4 ± 0.2^**a**^	–	14.50	***
22:1 n-9	0.3 ± 0.1^**c**^	4.1 ± 1.1^**a**^	1.2 ± 0.2^**b**^	–	14.33	***
18:1 n-9	1.5 ± 0.1^**c**^	12.7 ± 2.4^**a**^	4.0 ± 0.5^**b**^	0.3 ± 0.1^**d**^	14.12	***
18:2 n-6	6.0 ± 0.9^**a**^	2.5 ± 0.5^**c**^	4.0 ± 0.5^**b**^	3.4 ± 0.4^**bc**^	13.13	***
18:3 n-6	1.9 ± 0.1^**c**^	5.1 ± 1.3^**b**^	29.7 ± 1.1^**a**^	0.2 ± 0.1^**d**^	14.12	***
20:3 n-6	1.4 ± 0.2^**b**^	4.6 ± 0.8^**a**^	–	–	14.50	***
20:4 n-6	10.0 ± 3.3^**a**^	6.9 ± 1.9^**a**^	–	–	13.52	***
22:4 n-6	3.5 ± 0.7^a^	1.5 ± 0.4^b^	–	–	14.50	***
18:4 n-3	0.2 ± 0.1^c^	0.3 ± 0.1^c^	5.0 ± 0.2^b^	8.0 ± 0.7^a^	13.50	***
20:5 n-3	1.3 ± 0.1^**c**^	1.5 ± 0.7^**c**^	16.5 ± 2.2^**b**^	22.0 ± 1.7^**a**^	12.71	**
22:6 n-3	4.7 ± 1.7^**b**^	10.7 ± 2.6^**a**^	14.8 ± 2.0^**a**^	4.1 ± 0.9^**b**^	13.50	***

Data were analyzed using Kruskal-Wallis tests to determine the effect of compartment for each of 15 lipid species

*p<0.05

** *p*<0.01

***p<0.005.

Letters adjacent to values (mean±SD) represent statistically significant differences across compartments within a lipid species, as determined by Mann-Whitney post-hoc U tests (*p*<0.05).

“—” = not detected.

### Comparison of WE acyl chains

**Fig 3:**
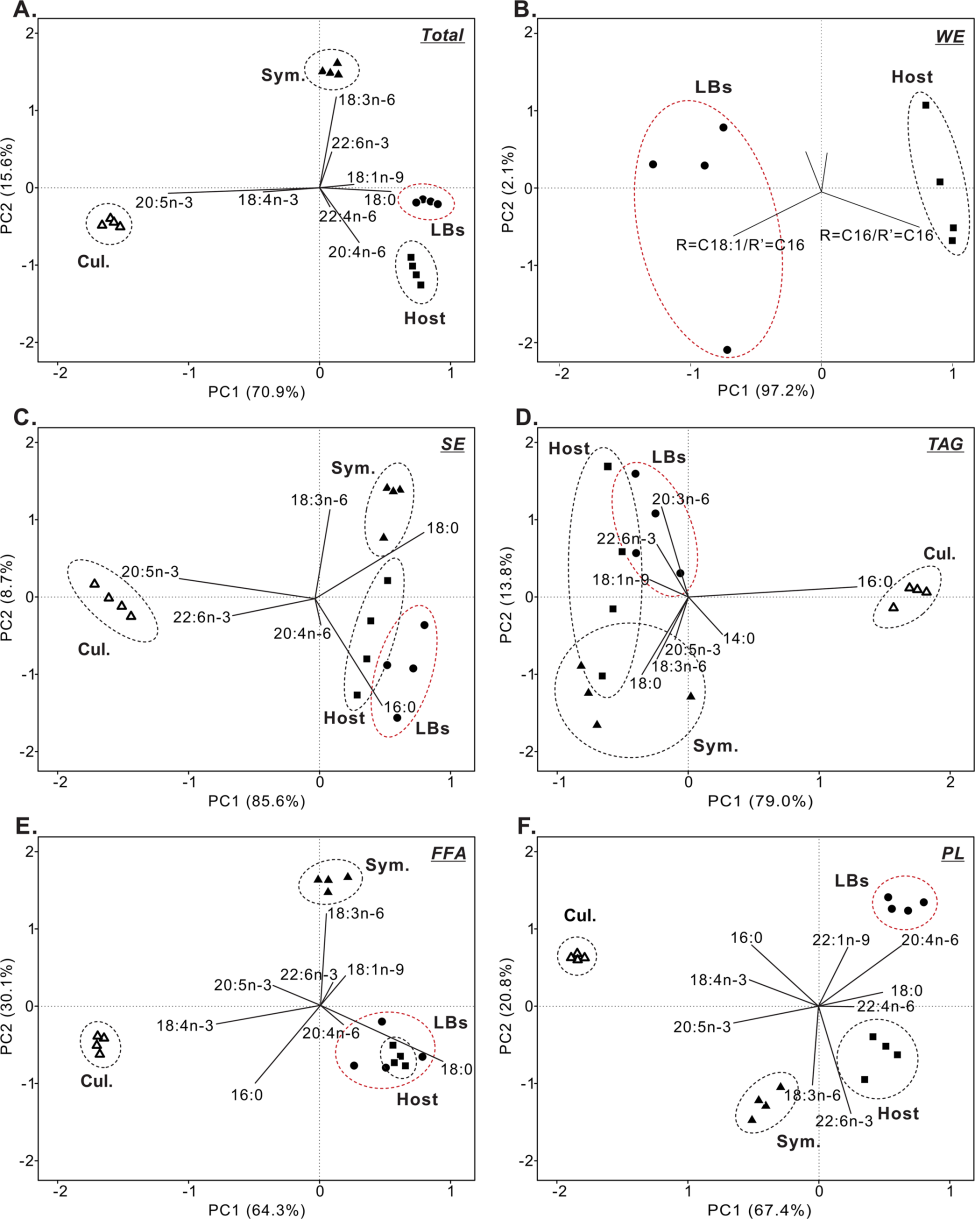
Principal components analysis of fatty acid moieties of what follows. (A) total lipids, (B) wax esters (WE), (C) sterol esters (SE), (D) triacylglycerols (TAG), (E) free fatty acids (FFA), and (F) phospholipids (PL) in coral host gastrodermal cells (“host”, solid squares), lipid bodies (“LBs”, solid circles), *in hospite Symbiodinium* (“Sym.”, solid triangles), and cultured *Symbiodinium* (“Cul.”, hollow triangles). Values represent mean±SD (n = 4).

As shown in Fig 2, WE were present only in host gastrodermal cells and LBs, and a total of four species of WE were identified with GC/MS (S3 Table and Fig 3B). LBs contained significantly higher concentrations of R = C14/R' = C16 (6.7±1.0 ng/μg protein), R = C18/R' = C16 (12.3±1.7 ng/μg protein), and R = C16/R' = C16 (20.0±2.7 ng/μg protein) in comparison to the host gastrodermal cells from which they were isolated. Furthermore, LBs contained high concentrations of R = C18:1/R' = C16 (13.4±3.4 ng/μg protein), which was undetectable in the host cells.

As shown in Fig 3B, PCA also demonstrated a separation of WE between LBs and gastrodermal cells. PC1 explained 97.2% of the variability, and PC2 explained 2.1%. The PC1 scores discriminated host gastrodermal cells, whose higher R = C16/R' = C16 concentrations contributed most significantly to the positive loading factors, from LBs, whose negative loading scores were reflective of a higher content of R = C18:1/R' = C16.

### Comparison of SE acyl chains

As shown in Fig 2, SE were measured at significantly higher concentrations in *in hospite Symbiodinium* (26.3±7.0 ng/μg protein) in comparison to host gastrodermal cells (8.5±0.3 ng/μg protein), LBs (9.8±2.4 ng/μg protein), and cultured *Symbiodinium* (19.2±4.9 ng/μg protein). PCA revealed that SE acyl chains of LBs and host gastrodermal cells were significantly distinct from those of *in hospite* and cultured *Symbiodinium* (Fig 3C). The concentration and composition of SE acyl chains differed significantly between *in hospite* and cultured *Symbiodinium*, as well (Fig 3C and S4 Table). In particular, according to the positive eigenvector for PC1, the cultured *Symbiodinium* possessed higher concentrations of 20:5n-3 (7.1±0.1 ng/μg protein) and 22:6n-3 (4.7±0.4 ng/μg protein) than LBs (0 and 0.4±0.2 ng/μg protein, respectively), host gastrodermal cells (0.3±0.1 and 0.5±0.3 ng/μg protein, respectively), and *in hospite Symbiodinium* (1.0±0.1 and 0.8±0.1 ng/μg protein, respectively). The fatty acid 18:3n-6 was only detected in *in hospite Symbiodinium* (4.4±1.5 ng/μg protein) and contributed to the negative eigenvector of PC2; it therefore enabled the separation of *in hospite Symbiodinium*, LBs, and coral host gastrodermal cells.

### Comparison of TAG acyl chains

As shown in Fig 2, TAG were the most abundant lipid species in both host gastrodermal cells and LBs. Fig 3D depicts the distributions of the correlations/concentrations along the two principal components (79.0% and 13.8%). Although TAG acyl chain pools of host gastrodermal cells were discernible, they overlapped significantly with the other two coral-derived fractions (i.e. LBs and *in hospite Symbiodinium*). The main differences in PC2 scores were observed for the species 18:0, 18:3n-6, and 20:5n-3, which were more concentrated in *in hospite Symbiodinium* than host coral gastrodermal cells and LBs. In contrast, the latter two cellular fractions tended to have higher concentrations of 20:3n-6, 22:6n-3, and 18:1n-9, and this allowed them to be distinguished from the *in hospite Symbiodinium* populations.

Moreover, cultured *Symbiodinium* could be readily distinguished from the three coral-derived fractions (see the difference in PC1 scores in Fig 3D). Along PC1, the acyl chain profiles of cultured *Symbiodinium* were clearly separated from those of coral host gastrodermal cells, LBs and *in hospite Symbiodinium*. The cultured *Symbiodinium* were characterized by a higher concentration of 16:0 (3.7±0.1 ng/μg protein) and a lower concentration of 18:1n-9 (0.1±0.0 ng/μg protein) relative to all other fractions (see also S5 Table). The opposite trend was observed for *in hospite Symbiodinium*, LBs, and host coral gastrodermal cells, which possessed higher concentrations of 18:0, 18:1n-9, 18:3n-6, 20:3n-6, and 22:6n-3 (see S5 Table).

### Comparison of FFA acyl chains

FFA were the most abundant lipid species in *in hospite Symbiodinium* samples (38.1±10.4 ng/μg protein), and FFA concentrations were significantly lower in LBs (26.8±7.5 ng/μg protein), cultured *Symbiodinium* (28.3±7.2 ng/μg protein), and host coral gastrodermal cells (18.8±0.6 ng/μg protein) (see Fig 2). Differences in the FFA profiles were better visualized by PCA (Fig 3E); the score plot explained 94.4% of the total variation present in the FA dataset, with PC1 accounting for 64.3% of the variation and PC2 accounting for 30.1%. For the *in hospite Symbiodinium* samples, 18:3n-6 was the most abundant FFA species (9.9±2.6 ng/μg protein, S6 Table). The acyl chains 18:0 and 20:4n-6 contributed the most negative scores for LBs and host gastrodermal cells and distinguished them from the *in hospite Symbiodinium*. The bi-plot differentiated LBs and host gastrodermal cells from *in hospite Symbiodinium* and cultured *Symbiodinium*. The acyl chains 16:0 (11.8±1.2 ng/μg protein across all four treatments), 18:4n-3 (5.9±0.6 ng/μg protein across all four treatments), and 20:5n-3 (2.9±0.9 ng/μg protein across all four treatments) were considered to serve as chemotaxonomic biomarkers for the FFA of cultured *Symbiodinium* in the PCA plot.

### Comparison of PL acyl chains

*in hospite Symbiodinium* possessed higher concentrations of PL (35.8±9.6 ng/μg protein) relative to host gastrodermal cells (25.7±0.8 ng/μg protein), cultured *Symbiodinium* (17.8±4.5 ng/μg protein), and LBs (10.0±2.5 ng/μg protein) (Fig 2). According to the multivariate ordination analysis, host gastrodermal cells, LBs, *in hospite Symbiodinium*, and cultured *Symbiodinium* were clearly separated by PCA (Fig 3F) on account of their PL acyl chains. The acyl chains that contributed most to the separation of groups along PC1 were 20:5n-3, 18:4n-3, 18:0, and 20:4n-6, which collectively explained 67.4% of the total variance. PC2 only explained 20.8% of the total variance. The cultured *Symbiodinium* contained the smallest amount of 18:0 (0.9±0.2 ng/μg protein) and the largest amounts of 20:5n-3 and 16:0 (4.0±0.8 and 6.1±1.0 ng/μg protein, respectively; S7 Table). The cultured *Symbiodinium* contributed most significantly to the positive score on PC1 and were clearly separated from the coral-derived fractions (i.e., LBs, host gastrodermal cells, and *in hospite Symbiodinium*). There were also clear differences between host coral gastrodermal cells, LBs, and *in hospite Symbiodinium*. The latter fraction was characterized by higher 18:3n-6 and 22:6n-3 concentrations. Furthermore, the 20:4n-6 and 22:1n-9 PL acyl chains were detected in LBs, which allowed for the acyl chain pools of the PLs in LBs to be distinguishable from those of host gastrodermal cells, *in hospite*, and cultured *Symbiodinium*. The acid 22:4n-6 from the coral host gastrodermal cells also contributed to the positive eigenvector of PC1.

### Data summary

**Fig 4:**
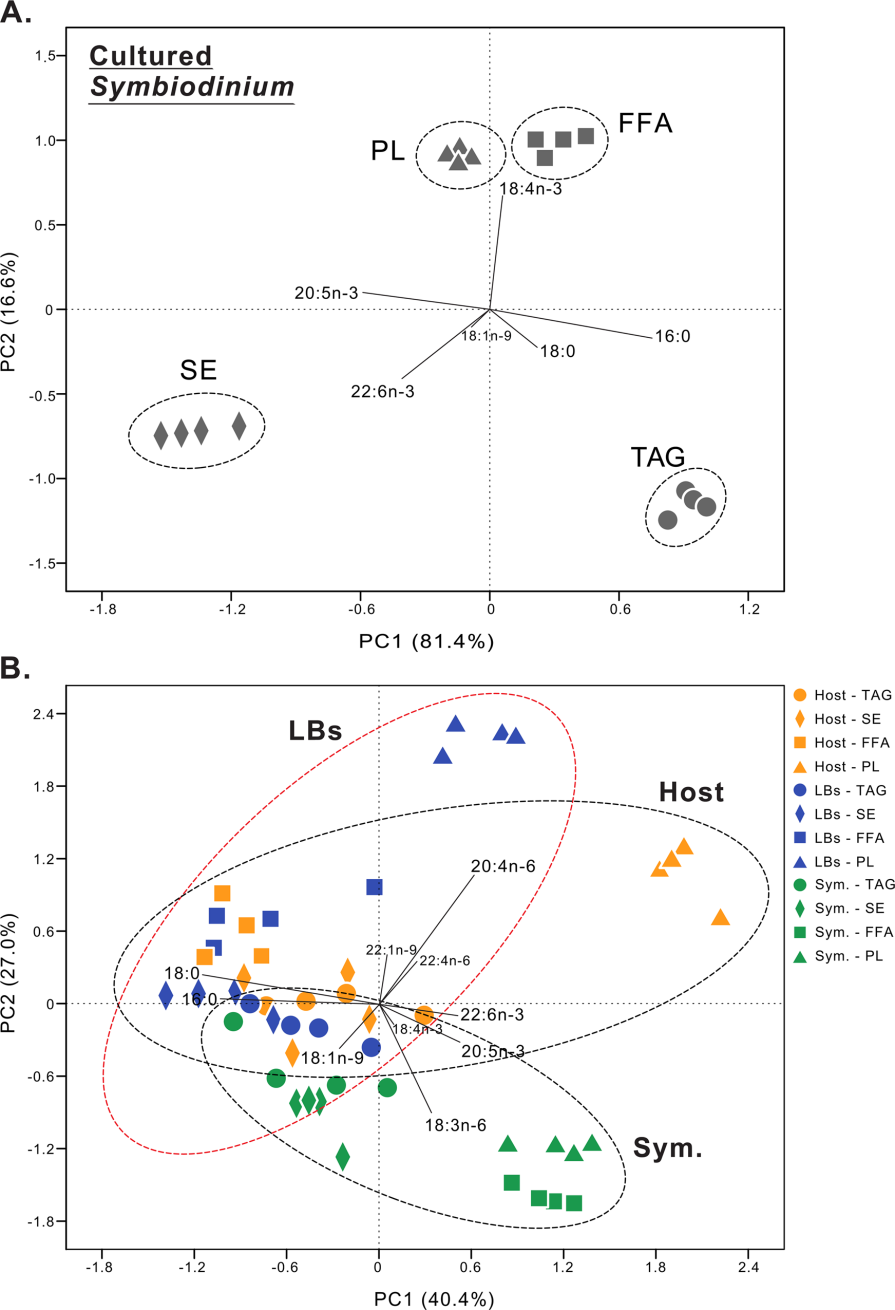
Principal components analysis (PCA) of the fatty acid moieties of what follows. (A) cultured *Symbiodinium*: sterol ester (SE; solid diamonds), triacylglycerol (TAG; solid circles), free fatty acid (FFA, solid squares), and phospholipid (PL, solid triangles). (B) PCA of fatty acid moieties of host gastrodermal cells (“host”), lipid bodies (“LBs”), and *in hospite Symbiodinium* (“Sym.”).

Amongst all lipid species within cultured *Symbiodinium* (Fig 4A), SE were most readily distinguished from the other primary species: TAG, FFA, and PL. The score plot explained 98.0% of the total variation present in the dataset, with PC1 accounting for 81.4% of the variation and PC2 accounting for 16.6%. The FFA and PL 18:4n-3 contributed most to the positive eigenvector of PC1. The differences in lipid acyl chains between host gastrodermal cells, LBs, *in hospite Symbiodinium*, and cultured *Symbiodinium* were also clearly separated by PCA (Fig 4B); PC1 explained 40.4% of the variation, with PC2 accounting for 27.0%. We found that most of the lipid classes (TAG, SE, and FFA, but not PLs) in the LBs tended to cluster with the host coral, rather than with the *in hospite Symbiodinium*. In particular, the SE acyl pools clustered more closely with those of other lipid species in the endosymbiotic compartments than with the cultured *Symbiodinium* populations. The acyl chain 20:4n-6 was distinguished as a chemotaxonomic biomarker for the host coral and LB fractions in the PCA plot, and 18:3n-6 contributed the most negative scores for *in hospite Symbiodinium*.

## Discussion

### Lipid pool distribution of coral host gastrodermal cells, LBs, and *in hospite Symbiodinium*

Previous studies have proposed that coral hosts actively alter the FFA compositions of their *in hospite Symbiodinium* populations [13, 23–26], but the molecular mechanisms underlying the regulation of these metabolic processes remain unclear [27]. In contrast, the transfer of FA synthesized by *Symbiodinium* to the coral host has been shown to significantly affect the latter’s FA pools, as deduced from studies in which “autotrophic” coral lipid profiles were found to differ from those of corals more adept at heterotrophic feeding [28]. However, the details of how each compartment modulates the lipid composition of the other has yet to be conclusively elucidated [29]. The present dataset reveals several notable differences in the lipid profiles of host coral gastrodermal tissues, LBs, *in hospite*, and cultured *Symbiodinium* and may therefore provide insight into this issue, as discussed below.

LBs are endosymbiotic-specific organelles that undergo dynamic changes in morphology and lipid composition over diel cycles, and they have been hypothesized to be the relay-station for lipid flow between the hosts and their *in hospite Symbiodinium* populations [8]. In this study, the lipid compositions of the host gastrodermal cells and LBs were more similar to each other than either was to *Symbiodinium*; this was mainly driven by the absence of WE in the latter compartment. We also found that the composition of acyl chains (with the important exception of those of the PL) in LBs was closer to that of the host coral than of the *in hospite Symbiodinium* (Fig 2). This may mean that, although it is likely that *Symbiodinium* do indeed provide their hosts with acyl chains necessary for the construction of more complex lipid species, the proximate origin of the lipids comprising the LBs is the coral host.

Previous studies have shown that biosynthesis of PUFA in marine invertebrates is a complicated and variable process [30–32]. The PUFA demonstrating the largest concentration differences among host gastrodermal cells, LBs, *in hospite*, and cultured *Symbiodinium* were 18:3n-6, 20:5n-3, and 22:6n-3 (Table 1). The PUFA are synthesized from a pivotal conversion of 18:1n-9 to 18:2n-6 catalyzed by Δ12-desaturase (i.e., the n-6 pathway), and then 18:2n-6 to 18:3n-3 by Δ15-desaturase (i.e., the n-3 pathway) [33]. Although some corals had higher concentrations of 18:4n-3, which has been shown to be a “specific” FA marker of *Symbiodinium* from certain milleporids, as well as and *Stylophora pistillata* [34], the *in hospite Symbiodinium* populations in this study possessed a high concentration of 18:3n-6. This was also shown in certain acroporids, all poritids studied to date, and some other hard corals [15, 24, 34–37]. In this study, the transfer of 18:3n-6 between the *in hospite Symbiodinium* and the host, or the activity of Δ5 and Δ6 desaturases in host gastrodermal cells, may have led to the high concentration of the arachidonic acid 20:4n-6 both in host gastrodermal cells and LBs (Table 1).

The FA composition of the *E*. *glabrescens* gastrodermal cells analyzed herein was characterized by high concentrations of 20:4n-6 and low 20:5n-3 concentrations. The 20:4n-6 has been previously detected in endosymbiotic corals [38], similar to *Clavularia viridis* and the soft coral *Sinularia* sp. [39–40]. Although previous study indicated that some corals (e.g., Milleporidae) accumulate high levels of 22:4n-6 and 22:5n-3, others (e.g., Acroporidae, Poritidae and Pocilloporidae) are more likely to produce 20:4n-6 and 20:5n-3, as was the case herein. 20:4n-6, 20:5n-3, 22:4n-6 and 22:5n-3 are all known to be key acids in the n-3 and n-6 PUFA pathways of marine invertebrates [34, 41–42]. Curiously, the former acid was not detected in either *in hospite* and cultured *Symbiodinium*, an observation supported by another study [26]. As a consequence, 20:4n-6 could serve as a marker for animal host tissue.

### The role of the host coral in LB lipid synthesis

WE were only present in the coral hosts, and they were highly concentrated in LBs (Fig 2). The fact that *Symbiodinium* did not produce WE seems to suggest that the WE of the LBs were derived from the host coral cells from which they were isolated, rather than having been translocated from the resident *Symbiodinium* populations. WE are the major lipid species in at least 30 species of marine animals, and some of their myriad functions include buoyancy, energy storage, and thermal insulation [43]. Although the role of WE in the coral-*Symbiodinium* endosymbiosis is currently unclear, previous studies of LBs may shed light on the origin of these lipids. Cheng and Russell [44–45] confirmed that WE synthase was localized to the membranes of the ER in animals using confocal light microscopy; such was also found to be the case in *Arabidopsis* [46]. These findings from other systems make it conceivable to postulate that the WE present within coral gastrodermal LBs are of host ER, and not of *Symbiodinium*, origin. However, not all LB WE are likely to be of host origin; palmityl oleate (R = C18:1/ R' = C16) was only found in LBs, not in the host gastrodermal cells. This may mean that palmityl oleate is produced endogenously within the LBs and could therefore serve as a LB marker.

Previous studies have found that lipid droplets originate from the ER in a variety of eukaryotic cells, in which neutral lipids (including TAG and SE) are synthesized between leaflets of the ER membranes and later bud off to form independent lipid droplets [47]. Peng *et al*. [8] identified the ER-specific chaperone Bip (GRP 78) in the coral LB proteome, potentially indicating a link between the host coral ER and the LBs. Furthermore, the concentrations of PL vary amongst organelles (e.g., the plasma membranes vs. the ER). There are normally high concentrations of PC and PE in the ER; however, sphingolipids and sterols are typically more abundant in the plasma membrane [48–49]. Our results revealed that the acyl composition of PL differed between host gastrodermal cells and LBs (Figs 3F and 4B and S7 Table), which is not surprising given the greater diversity of biological material (e.g., various organelles) in the host cell as a whole vs. the LBs. The striking differences in the acyl chain composition of the PL pools of LBs and the host gastrodermal cells may suggest that the LBs originate from the host ER.

### Lipid profiles of *in hospite* vs. cultured *Symbiodinium*

There were significant differences in the concentrations of the FA moieties of each lipid species between *in hospite* and cultured *Symbiodinium*. The most dominant FA in *in hospite Symbiodinium* were 16:0, 18:0,18:3n-6, 20:5n-3 and 22:6n-3; however, the major FA were 16:0, 18:4n-3 and 20:5n-3 in cultured *Symbiodinium* (Table 1). The predominance of the latter lipid species (i.e. 16:0, 18:4n-3 and 20:5n-3) has also been reported in other marine algae [50–51]. Furthermore, consistent to our result *in hospite Symbiodinium*, Bishop and Kenrick [24] have demonstrated that 18:3n-6, 20:5n-3, and 22:6n-3 were the major FA in the total lipid pools of *Symbiodinium* populations isolated from eight species of hard corals. It is possible that these differences in lipid profiles are related to metabolic differences between the *in hospite* and open ocean environments.

A major portion of the 18:2n-6 and 18:3n-3 pools in the host coral might have been supplied from external sources (e.g., phytoplankton and endosymbionts) [52–53], as it has been confirmed that the *Symbiodinium* populations within several reef-building corals have higher concentrations of PUFA in comparison to the host corals in which they were housed [13, 54]. This could be related to the fact that *Symbiodinium* are typically nitrogen limited *in hospite* [55–56]; Jiang *et al*. [57–58] also found that *in hospite Symbiodinium* from the sea anemone *Aiptasia pulchella* have higher proportions of PUFA than cultured *Symbiodinium*.

There was a distinct separation among acyl pools of each lipid species in cultured *Symbiodinium* (Fig 4A). However, SE clustered more closely with TAG in *in hospite Symbiodinium* populations (Fig 4B). SE are components of all eukaryotic membranes, and it has been suggested that they constitute a major storage pool of sterols. They are also important regulators of membrane fluidity and thus membrane properties and function [59–62]. We hypothesize that the acyl composition of *in hospite Symbiodinium* may depend not only on feeding status, but also on the light level/photoperiod; future studies are needed to determine whether such is indeed the case.

Wang *et al*. [63] found that *in hospite Symbiodinium* populations maintain high concentrations of FFA and PL and proposed that these lipids may be key players in the regulation of cell proliferation, recognition, and ultimately the success of the endosymbiosis. Our data indicate that FFA and PL concentrations are significantly higher in *in hospite* than cultured *Symbiodinium*. It could be that the host coral actively affects *Symbiodinium* lipid production, though further, radio-labeling-based experiments are needed to determine whether such is indeed the case [25–26].

## Conclusions

In this study, we found significant differences in the lipid profiles of LBs, coral host gastrodermal cells, and their *in hospite* (clade C) *Symbiodinium* populations. We also identified certain lipid species that were unique to the symbiotic relationship. Upon a collective assessment of the data, it appears that the direct translocation of final lipid products between LBs, host gastrodermal cells, and *in hospite Symbiodinium* documented in previous studies [23, 25, 29] cannot explain all of the compartmental differences. For instance, the LB lipids are likely to be of mixed origin, with some being from *Symbiodinium* and others from the host. This is based on the observation that the LB lipid composition was similar, but not equal to, that of the host gastrodermal cells in which they resided; the fact that some lipid species, though, were unique to LBs appears to suggest a degree of autonomous LB metabolism. Finally, there were significant differences in the lipid pools of the cultured (clade C) and *in hospite Symbiodinium* (clade C) populations, and this may suggest that the endosymbiotic lifestyle influences the lipid metabolism of this important *Symbiodinium* lineage. Direct examinations of the lipid flow between LBs, host gastrodermal cells, and their *in hospite Symbiodinium* communities, as well as how such fluxes differ over the diel cycle, will provide important insight towards an understanding of not only lipid metabolism, but also the molecular mechanisms underlying stable cnidarian-dinoflagellate endosymbioses. Further work should also seek to elucidate whether different lineages (i.e., clades) of *Symbiodinium* demonstrate similar lipid profile differences between the free-living and *in hospite* lifestyles.

## Supporting Information

S1 Table The gradient elution program for the first solvent system for HPLC separation of lipids. (DOCX)

S2 Table The gradient elution program of the second solvent system for HPLC separation of lipids. (DOCX)

S3 Table Wax ester (WE) concentrations (ng/μg protein) in the coral host gastrodermal cells and lipid bodies (LBs). (DOCX)

S4 Table Concentrations of sterol ester acyl chains in the coral host gastrodermal cells, lipid bodies (LBs), *in hospite Symbiodinium*, and cultured *Symbiodinium*. (DOCX)

S5 Table Concentrations of triacylglycerol acyl chains in the host coral gastrodermal cells, lipid bodies (LBs), *in hospite Symbiodinium*, and cultured *Symbiodinium*. (DOCX)

S6 Table Concentrations of free fatty acid acyl chains in the host coral gastrodermal cells, lipid bodies (LBs), *in hospite Symbiodinium*, and cultured *Symbiodinium*. (DOCX)

S7 Table Concentration of phospholipid acyl chains in the coral host gastrodermal cells, lipid bodies (LBs), *in hospite Symbiodinium*, and cultured *Symbiodinium*. (DOCX)
