# Supplementary material for: A Compartmental Comparison of Major Lipid Species in a Coral-Symbiodinium Endosymbiosis: Evidence that the Coral Host Regulates Lipogenesis of Its Cytosolic Lipid Bodies
Source: PLoS One. 2015 Jul 28;10(7):e0132519. doi: 10.1371/journal.pone.0132519 (PMC4517871; doi:10.1371/journal.pone.0132519)
Supplement: S1 Table — (DOCX) [file pone.0132519.s001.docx]

**S1 Table.** The gradient elution program for the first solvent system for HPLC separation of lipids.

| Time (min) |  | | | | Flow rate |
| --- | --- | --- | --- | --- | --- |
|  | Solvent | | | | (ml/min) |
|  | A (%) | B (%) | C (%) | D (%) |  |
| 0 | 100 | 0 | 0 | 0 | 1.0 |
| 4 | 100 | 0 | 0 | 0 | 1.0 |
| 7 | 50 | 50 | 0 | 0 | 1.0 |
| 10 | 0 | 100 | 0 | 0 | 1.0 |
| 15 | 0 | 85 | 15 | 0 | 1.0 |
| 20 | 0 | 83 | 17 | 0 | 1.0 |
| 25 | 0 | 75 | 25 | 0 | 1.0 |
| 30 | 0 | 40 | 60 | 0 | 1.0 |
| 32 | 0 | 30 | 50 | 20 | 1.0 |
| 35 | 0 | 25 | 35 | 40 | 1.0 |
| 40 | 0 | 20 | 30 | 50 | 1.0 |
| 45 | 0 | 25 | 75 | 0 | 1.0 |
| 50 | 0 | 45 | 55 | 0 | 1.0 |
| 55 | 0 | 65 | 35 | 0 | 1.0 |
| 60 | 0 | 85 | 15 | 0 | 1.0 |
| 65 | 0 | 100 | 0 | 0 | 1.0 |
| 70 | 50 | 50 | 0 | 0 | 1.0 |
| 75 | 100 | 0 | 0 | 0 | 1.0 |
