# Supplementary material for: A Compartmental Comparison of Major Lipid Species in a Coral-Symbiodinium Endosymbiosis: Evidence that the Coral Host Regulates Lipogenesis of Its Cytosolic Lipid Bodies
Source: PLoS One. 2015 Jul 28;10(7):e0132519. doi: 10.1371/journal.pone.0132519 (PMC4517871; doi:10.1371/journal.pone.0132519)
Supplement: S2 Table — (DOCX) [file pone.0132519.s002.docx]

**S2 Table.** The gradient elution program of the second solvent system for HPLC separation of lipids.

| Time (min) |  | | Flow rate |
| --- | --- | --- | --- |
|  | Solvent | | (ml/min) |
|  | A (%) | B (%) |  |
| 0 | 100 | 0 | 0.5 |
| 5 | 100 | 0 | 0.5 |
| 10 | 50 | 50 | 0.5 |
| 20 | 0 | 100 | 0.5 |
| 30 | 100 | 0 | 0.5 |
