# Supplementary material for: A Compartmental Comparison of Major Lipid Species in a Coral-Symbiodinium Endosymbiosis: Evidence that the Coral Host Regulates Lipogenesis of Its Cytosolic Lipid Bodies
Source: PLoS One. 2015 Jul 28;10(7):e0132519. doi: 10.1371/journal.pone.0132519 (PMC4517871; doi:10.1371/journal.pone.0132519)
Supplement: S3 Table — (DOCX) [file pone.0132519.s003.docx]

**S3 Table.** Wax ester **(**WE) concentrations (ng/μg protein) in the coral host gastrodermal cells and lipid bodies (LBs). Data from each lipid species were analyzed using the Kruskal-Wallis test to determine compartment differences within each lipid species, and letters adjacent to values (mean±SD) represent statistically significant differences across compartments (host gastrodermal cells vs. LBs) determined by Mann-Whitney U post hoc tests (*p*<0.05). “—“= not detected.

| WE | concentration (ng/μg protein) | | χ*^2^* value |
| --- | --- | --- | --- |
|  | Host gastrodermal cells | LBs |  |
|  |  |  |  |
| R=C14/R'=C16 | 2.3 ± 0.3**^b^** | 6.7 ± 1.0**^a^** | 5.33* |
| R=C16/R'=C16 | 10.6 ± 2.9**^a^** | 20.0 ± 7.2**^a^** | 4.08* |
| R=C18/R'=C16 | 3.2 ± 0.9**^b^** | 12.3 ± 1.7**^a^** | 5.33* |
| R=C18:1/R'=C16 | － | 13.4 ± 3.4 | 6.05* |
