# Supplementary material for: A Compartmental Comparison of Major Lipid Species in a Coral-Symbiodinium Endosymbiosis: Evidence that the Coral Host Regulates Lipogenesis of Its Cytosolic Lipid Bodies
Source: PLoS One. 2015 Jul 28;10(7):e0132519. doi: 10.1371/journal.pone.0132519 (PMC4517871; doi:10.1371/journal.pone.0132519)
Supplement: S4 Table — (DOCX) [file pone.0132519.s004.docx]

**S4 Table.** Concentrations of sterol ester acyl chains in the coral host gastrodermal cells, lipid bodies (LBs), *in hospite Symbiodinium*, and cultured *Symbiodinium*. Data were analyzed using a Kruskal-Wallis test (**p*<0.05, ** *p*<0.01, and ****p*<0.005) to determine the effect of compartment for each 15 lipid species, and letters adjacent to values (mean±SD) represent statistically significant differences across compartments within a lipid species, determined by Mann-Whitney U post hoc tests (*p*<0.05). “—“= not detected.

| Acyl chain | concentration (ng/μg protein) | | | | χ*^2^* value | *p* value |
| --- | --- | --- | --- | --- | --- | --- |
|  | Host gastrodermal cells | LBs | *in hospite Symbiodinium* | Cultured *Symbiodinium* |  |  |
|  |  |  |  |  |  |  |
| 14:0 | 0.2 ± 0.1**^b^** | 0.3 ± 0.0**^b^** | 1.3 ± 0.4**^a^** | － | 13.70 | *** |
| 16:0 | 2.9 ± 0.2**^c^** | 4.6 ± 0.3**^b^** | 8.7 ± 2.3**^a^** | 2.4 ± 0.4**^c^** | 13.50 | *** |
| 18:0 | 2.5 ± 0.7**^b^** | 3.5 ± 0.9**^b^** | 11.5 ± 1.8**^a^** | － | 13.70 | *** |
| 20:0 | － | － | － | － | － | － |
| 22:0 | － | － | － | － | － | － |
| 16:1 n-7 | － | － | 0.4 ± 0.1^a^ | － | 14.62 | *** |
| 20:2 n-9 | － | － | － | － | － | － |
| 22:1 n-9 | 0.2 ± 0.1^a^ | － | － | － | 14.62 | *** |
| 18:1 n-9 | 1.0 ± 0.6**^b^** | 1.2 ± 0.5**^b^** | 2.8 ± 0.7**^a^** | 1.2 ± 0.6**^b^** | 8.45 | * |
| 18:2 n-6 | 0.6 ± 0.3**^b^** | 0.3 ± 0.1**^b^** | 0.8 ± 0.5**^ab^** | 0.8 ± 0.1**^ab^** | 8.45 | * |
| 18:3 n-6 | － | － | 4.4 ± 1.5^a^ | － | 14.62 | *** |
| 20:3 n-6 | － | 0.2 ± 0.1^a^ | － | － | 14.62 | *** |
| 20:4 n-6 | 0.3 ± 0.2**^a^** | 0.3 ± 0.2**^a^** | － | － | 12.99 | *** |
| 22:4 n-6 | － | － | － | － | － | － |
| 18:4 n-3 | － | － | － | － | － | － |
| 20:5 n-3 | 0.3 ± 0.1**^c^** | － | 1.0 ± 0.1**^b^** | 7.1 ± 0.1**^a^** | 14.33 | *** |
| 22:6 n-3 | 0.5 ± 0.3**^bc^** | 0.4 ± 0.2^c^ | 0.8 ± 0.1**^b^** | 4.7 ± 0.4**^a^** | 11.71 | ** |
