# Supplementary material for: A Compartmental Comparison of Major Lipid Species in a Coral-Symbiodinium Endosymbiosis: Evidence that the Coral Host Regulates Lipogenesis of Its Cytosolic Lipid Bodies
Source: PLoS One. 2015 Jul 28;10(7):e0132519. doi: 10.1371/journal.pone.0132519 (PMC4517871; doi:10.1371/journal.pone.0132519)
Supplement: S5 Table — (DOCX) [file pone.0132519.s005.docx]

**S5 Table.** Concentrations of triacylglycerol acyl chains in the host coral gastrodermal cells, lipid bodies (LBs), *in hospite Symbiodinium*, and cultured *Symbiodinium*. Data were analyzed using a Kruskal-Wallis test (**p*<0.05, ** *p*<0.01, and ****p*<0.005) to determine the effect of compartment for each 15 lipid species, and letters adjacent to values (mean±SD) represent statistically significant differences across compartments within a lipid species, determined by Mann-Whitney U post hoc tests (*p*<0.05). “—“= not detected.

| Acyl chain | concentration (ng/μg protein) | | | | χ*^2^* value | *p* value |
| --- | --- | --- | --- | --- | --- | --- |
|  | Host gastrodermal cells | LBs | *in hospite Symbiodinium* | Cultured *Symbiodinium* |  |  |
|  |  |  |  |  |  |  |
| 14:0 | 0.5 ± 0.0**^c^** | 1.1 ± 0.2**^b^** | 2.7 ± 1.4**^a^** | 0.4 ± 0.0**^c^** | 13.79 | *** |
| 16:0 | 8.2 ± 1.3**^c^** | 22.8 ± 3.3**^a^** | 11.1 ± 0.9**^b^** | 3.7 ± 0.1**^d^** | 14.12 | *** |
| 18:0 | 6.8 ± 0.7**^c^** | 13.5 ± 1.3**^a^** | 10.7 ± 1.2**^b^** | 0.9 ± 0.1**^d^** | 14.12 | *** |
| 20:0 | 2.3 ± 0.1**^a^** | 0.3 ± 0.1**^b^** | － | － | 12.99 | *** |
| 22:0 | 0.3 ± 0.1^a^ | － | － | － | 14.62 | *** |
| 16:1 n-7 | － | － | 0.4 ± 0.3^a^ | － | 14.62 | *** |
| 20:2 n-9 | － | － | － | － | － | － |
| 22:1 n-9 | 1.0 ± 0.8**^a^** | 1.5 ± 0.3**^a^** | 0.8 ± 0.6**^a^** | － | 10.48 | * |
| 18:1 n-9 | 1.1 ± 0.2**^b^** | 6.9 ± 3.8**^a^** | 3.1 ± 1.7**^b^** | 0.1 ± 0.0**^c^** | 12.26 | ** |
| 18:2 n-6 | 0.2 ± 0.1**^b^** | 0.4 ± 0.1**^a^** | 0.5 ± 0.2**^a^** | － | 11.04 | * |
| 18:3 n-6 | 0.7 ± 0.2**^b^** | 0.5 ± 0.0**^b^** | 2.5 ± 1.5**^a^** | － | 10.48 | * |
| 20:3 n-6 | 2.1 ± 1.7**^b^** | 6.7 ± 2.3**^a^** | － | － | 14.12 | *** |
| 20:4 n-6 | 0.3 ± 0.1^a^ | － | － | － | 14.62 | *** |
| 22:4 n-6 | 0.1 ± 0.0**^a^** | 0.1 ± 0.0**^a^** | － | － | 13.52 | *** |
| 18:4 n-3 | 0.2 ± 0.1**^a^** | 0.3 ± 0.1**^a^** | 0.4 ± 0.2**^a^** | － | 10.90 | * |
| 20:5 n-3 | － | － | 1.3 ± 0.4^a^ | － | 14.62 | *** |
| 22:6 n-3 | 2.4 ± 1.4**^ab^** | 4.3 ± 1.9**^a^** | 1.0 ± 0.5**^b^** | － | 12.45 | ** |
