# Supplementary material for: A Compartmental Comparison of Major Lipid Species in a Coral-Symbiodinium Endosymbiosis: Evidence that the Coral Host Regulates Lipogenesis of Its Cytosolic Lipid Bodies
Source: PLoS One. 2015 Jul 28;10(7):e0132519. doi: 10.1371/journal.pone.0132519 (PMC4517871; doi:10.1371/journal.pone.0132519)
Supplement: S7 Table — (DOCX) [file pone.0132519.s007.docx]

**S7 Table.** Concentration of phospholipid acyl chains in the coral host gastrodermal cells, lipid bodies (LBs), *in hospite Symbiodinium*, and cultured *Symbiodinium*. Data were analyzed using a Kruskal-Wallis test (**p*<0.05, ** *p*<0.01, and ****p*<0.005) to determine the effect of compartment for each 15 lipid species, and letters adjacent to values (mean±SD) represent statistically significant differences across compartments within a lipid species, determined by Mann-Whitney U post hoc tests (*p*<0.05). “—“= not detected.

| Acyl chain | concentration (ng/μg protein) | | | | χ*^2^* value | *p* value |
| --- | --- | --- | --- | --- | --- | --- |
|  | Host gastrodermal cells | LBs | *in hospite Symbiodinium* | Cultured *Symbiodinium* |  |  |
|  |  |  |  |  |  |  |
| 14:0 | 0.3 ± 0.1**^b^** | 0.4 ± 0.2**^bc^** | 1.6 ± 0.5**^a^** | 0.5 ± 0.1**^b^** | 12.26 | ** |
| 16:0 | 4.0 ± 0.3**^c^** | 4.0 ± 0.9**^c^** | 13.4 ± 3.3**^a^** | 6.1 ± 1.0**^b^** | 12.20 | ** |
| 18:0 | 3.6 ± 0.1**^b^** | 3.6 ± 0.9**^b^** | 8.5 ± 2.3**^a^** | 0.9 ± 0.2**^c^** | 13.06 | *** |
| 20:0 | 0.3 ± 0.1^a^ | － | － | － | 14.62 | *** |
| 22:0 | 0.4 ± 0.1**^a^** | － | 0.9 ± 0.3**^a^** | － | 14.12 | *** |
| 16:1 n-7 | － | － | 0.2 ± 0.1^a^ | － | 14.62 | *** |
| 20:2 n-9 | 0.5 ± 0.0**^a^** | － | 0.7 ± 0.3**^a^** | － | 13.29 | *** |
| 22:1 n-9 | － | 2.0 ± 0.4^a^ | － | － | 14.62 | *** |
| 18:1 n-9 | 0.6 ± 0.1**^c^** | 3.6 ± 0.1**^a^** | 2.9 ± 1.4**^b^** | － | 14.12 | *** |
| 18:2 n-6 | 0.3 ± 0.1**^b^** | － | 0.6 ± 0.1**^a^** | － | 14.50 | *** |
| 18:3 n-6 | 0.4 ± 0.2**^b^** | － | 6.4 ± 1.6**^a^** | － | 14.33 | *** |
| 20:3 n-6 | 1.0 ± 0.3^a^ | － | － | － | 14.62 | *** |
| 20:4 n-6 | 4.2 ± 0.4**^a^** | 3.6 ± 1.0**^a^** | － | － | 13.11 | *** |
| 22:4 n-6 | 2.2 ± 0.2**^a^** | 0.8 ± 0.2**^b^** | － | － | 14.50 | *** |
| 18:4 n-3 | － | － | 2.1 ± 0.5**^b^** | 3.0 ± 0.5**^a^** | 14.12 | *** |
| 20:5 n-3 | 2.4 ± 0.4^b^ | 0.2 ± 0.2**^c^** | 6.8 ± 2.8**^a^** | 4.0 ± 0.8**^a^** | 13.45 | *** |
| 22:6 n-3 | 3.7 ± 0.8**^a^** | 0.4 ± 0.2**^b^** | 6.1 ± 2.4**^a^** | － | 14.33 | *** |
